# Metagenomic Insights into Pigeon Gut Microbiota Characteristics and Antibiotic-Resistant Genes

**DOI:** 10.3390/biology14010025

**Published:** 2025-01-01

**Authors:** Wei Dai, Haicong Zhu, Junhong Chen, Hui Chen, Dingzhen Dai, Jian Wu

**Affiliations:** 1 School of Animal Science and Food Engineering, Jinling Institute of Technology, Nanjing 210038, China; daiwei@jit.edu.cn (W.D.); a3023450661@163.com (H.Z.); chenjunhong@jit.edu.cn (J.C.); chenhui5410@jit.edu.cn (H.C.); daimingrui@jit.edu.cn (D.D.); 2 Department of Bioinformatics, Nanjing Medical University, Nanjing 211166, China

**Keywords:** pigeon, metagenomics, gut microbiota, antibiotic-resistant genes

## Abstract

Background: Antibiotics were extensively used in the pigeon breeding industry previously to promote growth and prevent disease, leading to the spread of antibiotic-resistant genes (ARGs) in gut microbes, which has become a major public health concern. Methods: A metagenomic analysis was performed to investigate the gut microbial communities and ARGs in young and older pigeons in Nanjing, Jiangsu Province, China. Results: There were obviously distinct gut microbiota and functional compositions between young and older pigeons. Both Pseudomonadota and Uroviricota were dominant in young and older pigeons. Although sharing 24 gut microbiota phyla between young and older pigeons, Bacillota and Pseudomonadota were the dominant microbial phyla in them, respectively. Besides the shared metabolic pathways and biosynthesis of secondary metabolites, biosynthesis of amino acids was the most abundant Kyoto Encyclopedia of Genes and Genomes (KEGG) function in young pigeons, while microbial metabolism in diverse environments was abundant in older pigeons. A total of 142 ARGs conferring multidrug resistance, tetracycline, and aminoglycoside resistance were identified; the most abundant gene in young pigeons was tetracycline-tetW, while in older pigeons, it was multidrug-acrB. Conclusions: Our findings revealed significant differences in the gut microbial communities and ARGs between young and older pigeons. This study enhances our understanding of pigeon gut microbiota and antibiotic resistomes, contributing to knowledge-based sustainable pigeon meat production.

## Simple Summary

Antibiotics were extensively used in the pigeon breeding industry previously, leading to the spread of antibiotic-resistant genes (ARGs) and their deposition in gut microbes, which has become a major public health concern. In this study, we found the distinct gut microbiota and functional compositions between young and older pigeons in Nanjing, Jiangsu Province, China, by using a metagenomic analysis. Streptococcus and Escherichia were the most abundant genera in young and older pigeons, respectively. The gene ontology (GO) analysis highlighted the significant enrichment of integral components of the membrane, ATP binding, and DNA binding in pigeon gut microbiota. The CAZy functional classification showed that glycoside hydrolases, glycosyl transferases, and carbohydrate esterases were the most abundant in pigeon gut microbes. Moreover, 142 ARGs conferring multidrug resistance, tetracycline, and aminoglycoside resistance were identified, representing more than 70% of the fecal resistomes; the most abundant gene in young pigeons was tetracycline-tetW, while in older pigeons, it was multidrug-acrB. Additionally, we found the highest abundance of resistance genes in older pigeons, indicating a high level of antibiotic resistance.

## 1. Introduction

The intestine is where the body carries out digestion, absorption, and nutrient transportation. The gut microbiota possesses rich biodiversity and plays a crucial role in maintaining the physiological balance of the intestine [1]. It helps the host with nutrient absorption, energy intake, regulation of metabolic processes, and promotion of intestinal development. Some gut microbiota also participate in immune regulation and enhance individual resistance [2]. Therefore, the characteristics of gut microbiota are closely related to the health and diseases of the host [3]. In recent years, with the intensive use of antibiotics in the breeding industry to promote growth and prevent diseases, an increasing number of antibiotic-resistant bacteria (ARB) have been found in animal stools, as the intestine cannot completely digest these antibiotics. This has placed significant pressure on Chinese poultry production [4]. More importantly, the use of antibiotics can have a genetic selection effect on microorganisms, leading to the enrichment of antibiotic-resistance genes (ARGs). The animal gut microflora is found to be a vast reservoir for ARGs. When ARGs are exposed and spread, they become a major worldwide health issue and may cause ecological damage, which requires attention [5].

The gut microbiome is considered the host’s second genome [6], playing an important role in maintaining host metabolism, immunity, and physiological functions, thus requiring further characterization [7]. Traditional microbiology research has always relied on bacterial isolation and cultivation techniques; however, approximately 85–99% of gut microflora cannot be cultivated in the laboratory [8]. This limitation hinders a deep understanding of bacterial functions, including those associated with antibiotic resistance, making it extremely difficult to fully utilize microbial diversities for disease detection and treatment research [9]. PCR or 16S rRNA gene sequencing technology makes it difficult to decipher the functional capabilities of gut microflora when focusing on limited segments of a gene [10].

Recently, with the development of sequencing platforms such as next-generation sequencing (NGS) and the rapid decline in sequencing costs, substantial research into the diversity and function of gut microbiota from various livestock animals has become possible [11]. Metagenomics is the study of genetic materials from environmental or host-associated microbiota to identify microbial diversity and function [12]. Compared to 16S rRNA gene sequencing, metagenomics can produce assemblies of higher resolution and less abundant taxa and their functional potential can be identified, which can reveal more microbial diversity within and between samples [13,14]. Therefore, metagenomics is increasingly used to study gut microbiota and the ARGs in livestock animals, helping to assess the risk of ARGs to human health [15].

Large-scale pigeon breeding in China began in the early 1980s. After nearly 40 years of effort and accumulation, the pigeon farming industry has gradually established a set of mature and complete breeding technology systems in the country [16]. Pigeon is the fourth-largest poultry product in China, following chickens, ducks, and geese. There are approximately 40 million pairs of breeding pigeons in China, and more than 600 million commercial pigeons are sold annually [17]. Pigeon meat and eggs are consumed as nutritional food and are gaining popularity among consumers in Europe, the United States, and Japan [18]. Additionally, pigeons are also popular as racing and fancy birds in many areas of the world. However, with the continuous expansion of pigeon breeding, pigeon infectious diseases are becoming increasingly serious. Since China banned the use of antibiotics as growth promoters in 2020, there is limited information on the diversity and functions of ARGs in pigeon gut microbiota and their impact on the growth and health of the host, as well as on human health [19]. The gut microbiota of pigeons is a key factor in maintaining host health and resisting diseases. It is influenced by various factors, such as nutritional ratios, disease occurrence, and the maturity of the pigeons themselves. Therefore, identifying gut microbiota is of great significance for the prevention of diseases and the promotion of sustainable development in the pigeon breeding industry.

In this study, we perform a comprehensive investigation of pigeon gut microbiota using metagenomic technology. We analyze the differences in gut microbiota between young and older pigeons, aiming to further enrich the data on the gut microbial diversity of pigeons. Additionally, the ARGs in pigeons were analyzed to provide early warning and strong support for the healthy breeding of pigeons.

## 2. Materials and Methods

### 2.1. Source of Samples

In this study, 15 fecal samples from young pigeons (ranging from 2 to 5 months old), weighing from 250 g to 450 g, were collected from a pigeon breeding farm in Luhe District, Nanjing. Additionally, 15 fecal samples from older pigeons (ranging from 5 months to 2 years old), weighing from 1000 g to 1500 g, were collected from a pigeon breeding farm in Gaochun District, Nanjing. The collected samples were transported to the laboratory on ice and stored at −80 °C until further analysis.

### 2.2. DNA Extraction and Sequencing

DNA was extracted from each pigeon fecal sample using a TIANamp Stool DNA Kit (TIANGEN, Beijing, China). A 1% agarose gel was used to analyze the degree of DNA degradation. The concentration and quality of the DNA were measured using a NanoDrop 2000 spectrophotometer (ThermoFisher, Waltham, MA, USA). High-quality metagenomic DNA (DNA concentration > 10 ng/μL and amount > 1 μg) was then used for library construction with the NEBNext Ultra DNA Library Prep Kit for Illumina (NEB, Ipswich, MA, USA). The metagenomic DNA library from pigeon fecal samples was subsequently sequenced on the Illumina HiSeq 2500 platform with 150 bp paired-end reads (PE150). The DNA libraries from the young pigeon group were mixed into one sequencing sample, referred to as YP, and the DNA libraries from the older pigeon group were mixed into one sequencing sample, referred to as OP.

### 2.3. Quality Control and Metagenome Assembly

FASTQ (version 0.36) was used to perform quality control and host filtering on the raw reads obtained from metagenomic sequencing data [20]. Bowtie2 (version 2.1.0) was used to align the raw reads to the host genome (*Columba livia*, NO. SRA054954, http://dx.doi.org/10.5524/100007) [21], and reads with high alignment similarity from the host genome or contamination were removed. After obtaining high-quality clean reads, MEGAHIT (version 1.1.2) was used to splice and assemble the clean reads into contigs with default parameters based on the overlap relationships between reads. Then, the clean reads of each sample were aligned to the assembled contigs using Bowtie2 to obtain unmapped PE reads; SPAdes was used to perform mixed splicing on the unaligned reads; for the contigs generated by the two splicing assemblies, sequences less than 500 bp were filtered.

### 2.4. Gene Prediction and Gene Set Construct

The open reading frames (ORFs) of contigs longer than 100 bp were predicted using Prodigal (version 2.60) with a metagenomic model [22]. The predicted gene sequences of all samples were clustered using CD-HIT (version 4.6) by maintaining a clustering threshold of 95% and a coverage threshold of 90%. The longest gene was selected as the representative sequence for each cluster to construct a non-redundant gene set. Then, Bowtie2 was used to compare the clean reads of each sample with the non-redundant gene set, and SAMtools (version 0.1.18) was used to count the number of compared reads and summarize it into a gene abundance.

### 2.5. Microbiota Taxonomy Annotation

DIAMOND (version 0.8.20) was used to perform a BLASTP homology alignment between gene set protein sequences and NCBI non-redundant protein sequences (Nr) database to obtain functional annotations and homologous species information (E-value < 1 × 10^−5^, score > 60). The lowest common ancestor (LCA) algorithm was used to obtain the number of genes and abundance information for each sample at each taxonomic hierarchy (kingdom, phylum, class, order, family, genus, and species).

### 2.6. Functional Annotation

The Kyoto Encyclopedia of Genes and Genomes (KEGG) (http://www.kegg.jp, accessed on 10 January 2024), the evolutionary genealogy of genes Non-supervised Orthologous Groups (eggNOG) (http://eggnogdb.embl.de/, accessed on 10 January 2024), and Carbohydrate-Active enZymes (CAZy) (http://www.cazy.org/, accessed on 10 January 2024) are the most frequently used databases for studying the functional properties of microbial populations [23]. The metagenomic reads from each sample were mapped to the eggNOG (version 4.0) [24] and KEGG databases [25] with default parameters (E-value ≤ 1 × 10^−5^) using DIAMOND software, and the highest-scoring annotated hit was selected.

The protein sequences of the gene set were aligned with the Uniprot database using DIMOND software to obtain the gene ontology (GO) items (E-value < 1 × 10^−5^, score > 60). DIAMOND software was also used to perform BLASTP homology alignment between gene set protein sequences and the SEED database to obtain functional annotations and homologous species information (E-value < 1 × 10^−5^, score > 60). At the same time, the SEED annotation information of genes based on the functional classification hierarchy of the SEED subsystem was obtained.

HMMER3 software (version 3.1b1) was used to compare the protein sequences of non-redundant genes with the CAZy database (version 6.0) to identify carbohydrate-active enzymes in the genome.

### 2.7. Antibiotic Resistance Genes Annotation

The non-redundant gene set of the gut microbiome was further searched against the Comprehensive Antibiotic Resistance Database (CARD) to assess the presence of ARGs, using DIAMOND software with E-value < 1 × 10^−5^ and score > 60 [26].

### 2.8. Statistical Analysis and Visualization

Alpha diversity, including Chao1, Shannon–Wiener, and Simpson indices, was calculated and plotted using the Tutools platform (https://www.cloudtutu.com, accessed on 10 January 2024). Based on the relative abundance of species or functions, all sample structure component graphs were compared together to draw a bar graph using R-ggplot2 software (version 3.4.4). Due to the large number of species classifications, only the top 10 species or functional classifications in abundance were selected and included in the bar graph. A bar chart, collinear relationship diagram, and heatmap were all drawn with the R package (version 3.3).

## 3. Results

### 3.1. Metagenomic Sequencing and Gene Prediction

The metagenomic sequencing generated 38,220,082 raw reads, corresponding to 5,733,012,300 bases, from the intestinal stool of young pigeons (referred to as the YP sample) and 40,093,458 raw reads, corresponding to 6,014,018,700 bases, from the intestinal stool of older pigeons (referred to as the OP sample). Low-quality reads were filtered from the YP and OP sample datasets to obtain clean reads. After filtering, 36,035,534 clean reads with a retention rate of 94.28% were retained from the YP sample, and 37,528,722 clean reads with a retention rate of 93.60% were retained from the OP sample (Table S1).

After assembling and predicting the sequencing data, a total of 147,020 genes were identified between the YP and OP samples. Gene alpha diversity analysis showed greater richness and diversity in older pigeons compared to young pigeons. The Chao1, ACE, and Shannon indices in older pigeons were 81,253, 81,679, and 8.9, respectively, which were much higher than those in young pigeons, where the Chao1, ACE, and Shannon indices were 69,101, 69,477, and 7.6, respectively (Table S2).

### 3.2. Composition of Gut Microbial Communities

**Figure 1:**
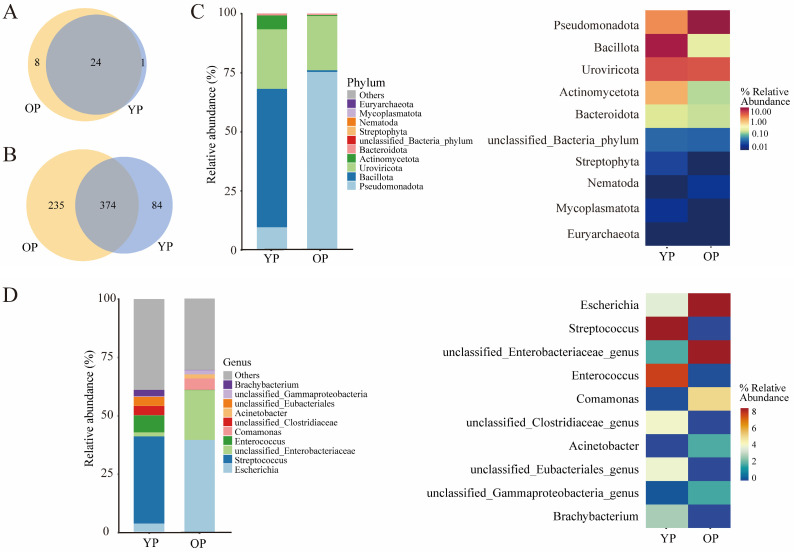
Taxonomic annotation of gut microbiota between young pigeon and older pigeon. Schemes follow the same formatting. (**A**) Venn diagram showing the number of common and unique phyla among pigeons. (**B**) Venn diagram showing the number of common and unique genera among pigeons. (**C**) Relative abundance of intestinal microbiota at the phylum level. (**D**) Relative abundance of gut microbiota at the genus level.

The assembled gene sets were aligned against the non-redundant protein database to determine the microbial community composition and relative abundance. The distributions of individual samples at the phylum and genus levels were investigated. A Venn analysis indicated that 1 and 8 unique phyla were identified in the gut microbiota of young and older pigeons, respectively (Figure 1A). A total of 84 and 235 unique genera were identified in the intestinal microbiota of young and older pigeons, respectively (Figure 1B). Among them, Bacillota, Uroviricota, and Pseudomonadota were the dominant microbial phyla in young pigeons, accounting for 58.58%, 25.19%, and 9.62%, respectively, while Pseudomonadota and Uroviricota were the dominant microbial phyla in older pigeons, accounting for 75.29% and 22.93%, respectively (Figure 1C). Streptococcus, Enterococcus, and unclassified_Clostridiaceae were the dominant microbial genera in young pigeons, accounting for 37.39%, 7.30%, and 3.96%, respectively. The dominant Streptococcus would result in meningitis, septicemia, and streptococcal toxic shock syndrome, which is mainly spread by the house mouse (Figure S1, Table S3). While Escherichia, unclassified_Enterobacteriaceae, and Comamonas were dominant microbial genera in older pigeons, accounting for 39.53%, 21.28%, and 4.88%, respectively (Figure 1D and Figure S2). The dominant Escherichia coli would result in urinary tract infections, which are also spread by the house mouse (Figure S1, Table S4). Furthermore, heatmap analysis indicated that the abundance of Bacillota was higher than that of other phyla in young pigeons, while the abundance of Pseudomonadota was higher than that of other phyla in older pigeons (Figure 1C). Streptococcus was more abundant than other genera in young pigeons, while Escherichia was more abundant than other genera in older pigeons (Figure 1D).

### 3.3. Gene Function of the Gut Microbes

**Figure 2:**
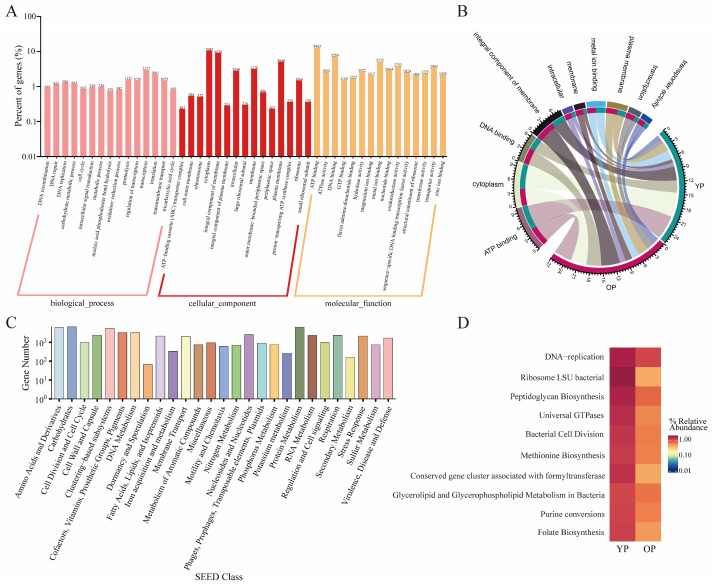
Gene function annotation of gut microbiota between young pigeon and older pigeon. (**A**) Statistical map of GO term-related functional genes. The *X*-axis is the classification content of the GO term, and the *Y*-axis is the relative percent of the corresponding functional genes. (**B**) Circos plot of relative abundance of GO term. The right side of the circle indicates group information, and the left side indicates GO term information; the outer circle is the ideogram scale of the distribution of unique genes, and the inner circle represents different groups. (**C**) Histogram of SEED functional gene function classification. The *X*-axis is the classification content of SEED; the *Y*-axis is the relative number of the corresponding functional genes. (**D**) Heatmap representation of SEED level 3 abundance of the top 10 dominant terms.

The gut microbial functions in young pigeons and older pigeons were annotated based on the GO and SEED databases. Gene function classification analysis revealed that in pigeon gut microbes, transcription, translation, and proteolysis were the main GO functions in biological processes; cytoplasm, integral component of membrane, and plasma membrane were the main GO functions in cellular components; and ATP binding, DNA binding, and metal ion binding were the main GO functions in molecular functions (Figure 2A). The overall presence of GO terms is depicted in a Circos plot, which shows that the predominant types of GO functions in both young and older pigeons were integral components of membrane, ATP binding, and DNA binding (Figure 2B). SEED annotation results indicated that carbohydrates, amino acids and derivatives, protein metabolism, clustering-based subsystems, cofactors, vitamins, prosthetic groups, and pigments were the most prevalent in terms of gene numbers in both young and older pigeon gut microbes (Figure 2C). Additionally, the heatmap of the SEED category level 3 groups showed that the abundance of ribosome LSU bacterial proteins, DNA replication, and universal GTPases was higher than that of other gene functions in the young pigeon sample, accounting for 1.72%, 1.66%, and 1.55%, respectively. In contrast, the abundance of DNA replication (1.19%), peptidoglycan biosynthesis (0.84%), and glycerolipid and glycerophospholipid metabolism in bacteria (0.74%) was higher than that of other functions in the older pigeon sample (Figure 2D).

### 3.4. Functional Pathway Annotation

The functional pathways of gut microbiota were annotated based on the KEGG and eggNOG databases.

**Figure 3:**
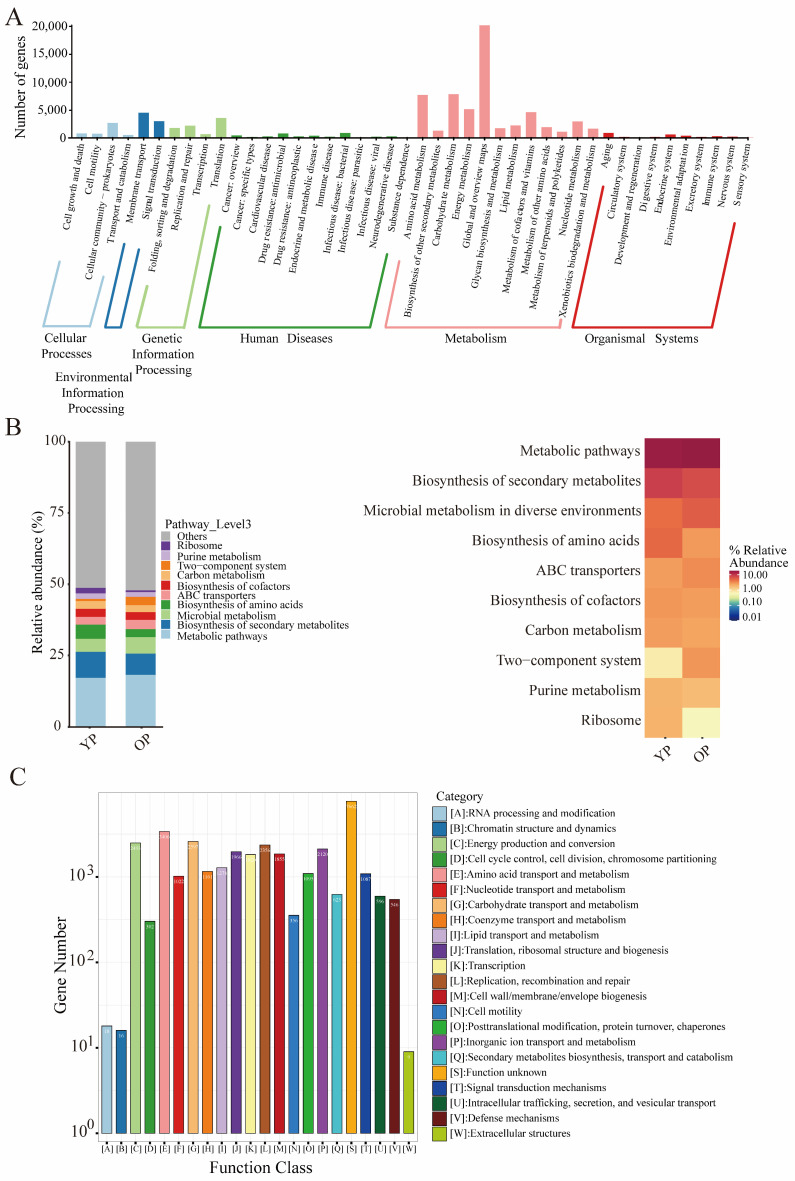
Functional pathway annotation of gut microbiota between young pigeon and older pigeon. (**A**) Statistical map of KEGG metabolic pathway-related functional genes at level 2. The *X*-axis is the classification content of the KEGG pathway at level 1 and level 2; the *Y*-axis is the relative number of the corresponding functional genes. (**B**) Relative abundance of KEGG pathway at level 3. (**C**) Histogram of eggNOG functional gene function classification. The *X*-axis is the classification content of eggNOG; the *Y*-axis is the relative number of the corresponding functional genes.

KEGG pathway classification analysis indicated that the functional pathways of cellular community prokaryotes, membrane transport, translation, infectious disease bacteria, global and overview maps, and endocrine system pathways had relatively high numbers of genes in the cellular processes, environmental information processing, genetic information processing, human disease, metabolism, and organismal system classifications of the pigeon gut microbiota, respectively (Figure 3A). Furthermore, metabolic pathways (17.19%), biosynthesis of secondary metabolites (9.07%), and biosynthesis of amino acids (4.98%) were the most abundant KEGG functions in the young pigeon sample; metabolic pathways (18.22%), biosynthesis of secondary metabolites (7.4%), and microbial metabolism in diverse environments (5.78%) were the most abundant KEGG functions in the older pigeon sample (Figure 3B). Heatmap analysis indicated that the abundance of metabolic pathways was higher than that of other KEGG functions in both young and older pigeon gut microbiota (Figure 3B). Additionally, eggNOG annotation results showed that, besides unknown functions, amino acid transport and metabolism, carbohydrate transport and metabolism, energy production and conversion, and replication, recombination, and repair were the main functions of pigeon gut microbes, with the number of genes in these eggNOG functions being 3406, 2597, 2493, 2356, and 2120, respectively (Figure 3C).

### 3.5. Characteristics of Carbohydrate Metabolism in Gut Microbes

To investigate the characteristics of carbohydrate metabolism in gut microbes between young and older pigeons, gut microbial functions were annotated based on the CAZy databases.

**Figure 4:**
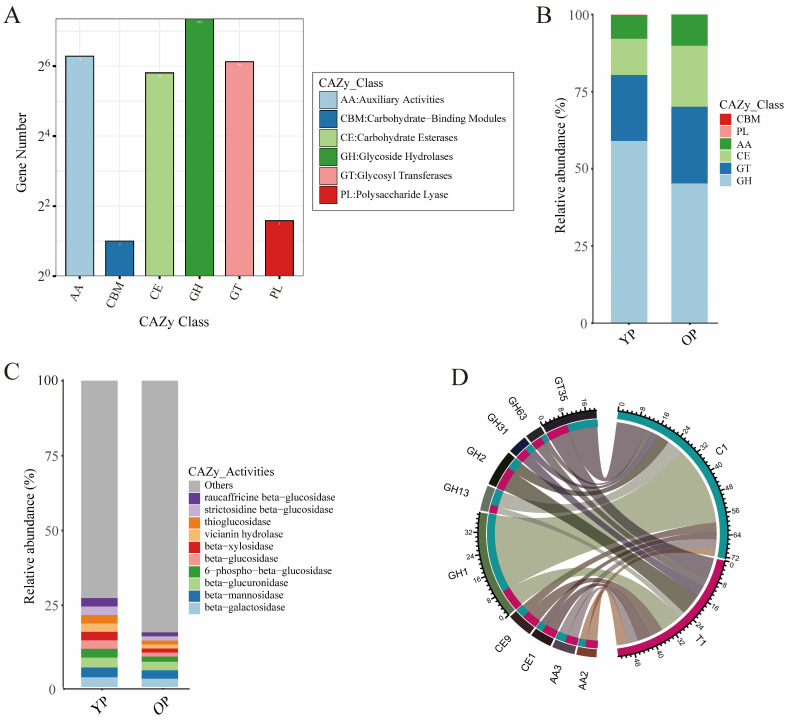
Carbohydrate enzyme characteristics of gut microbiota between young pigeon and older pigeon. (**A**) Histogram of CAZy functional gene function classification. The *X*-axis is the classification content of CAZy; the *Y*-axis is the relative number of the corresponding functional genes. (**B**) Relative abundance of CAZy term at the class level. (**C**) Relative abundance of CAZy term at activity level. (**D**) Circos plot of relative abundance of CAZy classification. The right side of the circle indicates group information, and the left side indicates CAZy classification information; the outer circle is the ideogram scale of the distribution of unique genes, and the inner circle represents different groups.

The results indicated that 164 genes were related to glycoside hydrolases (GH), 78 genes to auxiliary activities (AA), 70 genes to glycosyl transferases (GT), 56 genes to carbohydrate esterases (CE), 3 genes to polysaccharide lyase (PL), and 2 genes to carbohydrate-binding modules (CBM) in pigeon gut microbes (Figure 4A). The CAZy functional classification analysis indicated that glycoside hydrolases, glycosyl transferases, and carbohydrate esterases were the most abundant CAZy classes in both young and older pigeon gut microbes (Figure 4B). The beta-galactosidase, beta-mannosidase, and beta-glucuronidase were the most abundant CAZy activities in both young and older pigeon gut microbes (Figure 4C). Further, the overall presence of CAZy terms is depicted as a Circos plot, which shows that the predominant types of CAZy functions in young pigeons were GH1, GT35, and GH13, while the predominant types in older pigeons were GH2, GT35, and GH1 (Figure 4D). GH1, GT35, and GH13 were the most abundant CAZy groups in young pigeon gut microbes, accounting for 31.06%, 11.8%, and 6.5%, respectively; GH2 (8.45%), GT35 (8.06%), and GH1 (8.05%) were the most abundant CAZy groups in older pigeon gut microbes.

### 3.6. Abundance and Diversity Analysis of ARGs in Gut Microbes

**Figure 5:**
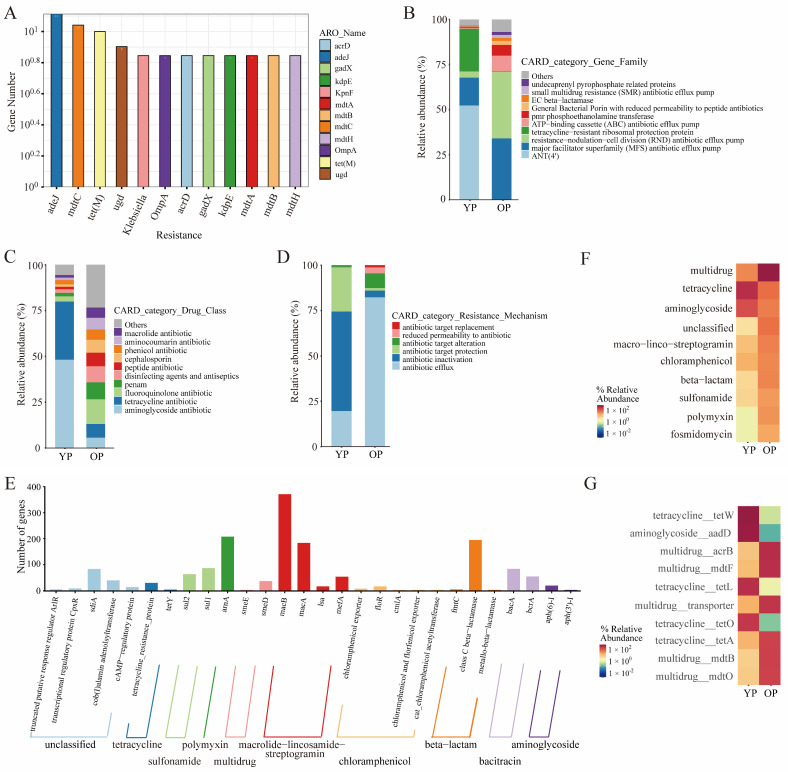
The abundance of antibiotic-resistant genes of gut microbiota between young pigeons and older pigeons. (**A**) Histogram of antibiotic-resistant gene types. The *X*-axis is the classification content of antibiotic-resistant genes; the *Y*-axis is the relative number of the corresponding genes. (**B**) Relative abundance of ARGs at the gene family level. (**C**) Relative abundance of ARGs at drug class level. (**D**) Relative abundance of ARGs at the resistance mechanism level. (**E**) Statistical map of ARG types. The *X*-axis is the classification content of ARGs; the *Y*-axis is the relative number of the corresponding ARGs. (**F**) Heatmap representation of ARG types abundance of the top 10 dominant terms. (**G**) Heatmap representation of ARGs subtypes abundance of the top 10 dominant terms.

In total, 142 ARGs were detected in the pigeon gut microbiota compared to the CARD database. The relative abundance of the top 12 ARGs is displayed as a histogram, with adeJ, mdtC, tet(M), and udg being the dominant resistant types (Figure 5A). The identified ARGs were further categorized based on gene family, drug class, and resistance mechanism. The results indicated that ANT (4’) (52.29%), tetracycline-resistance ribosomal protection protein (23.60%), and major facilitator superfamily (MFS) antibiotic efflux pump (15.45%) were the most abundant gene family types in the young pigeon sample, while resistance–nodulation–cell division (RND) antibiotic efflux pump (37.04%), major facilitator superfamily (MFS) antibiotic efflux pump (33.97%), and ATP-binding cassette (ABC) antibiotic efflux pump (8.65%) were the most abundant gene family types in the older pigeon sample (Figure 5B). Aminoglycoside antibiotics (48.34%), tetracycline antibiotics (31.59%), and fluoroquinolone antibiotics (2.83%) were the most abundant drug-resistant types in young pigeons; fluoroquinolone antibiotics (13.35%), penam (9.25%), and disinfecting agents and antiseptics (8.78%) were the most abundant drug-resistant types in older pigeons (Figure 5C). Antibiotic inactivation (54.83%), antibiotic target protection (24.34%), and antibiotic efflux (19.63%) were the most abundant resistance mechanism types in young pigeons; antibiotic efflux (82.26%), antibiotic target alteration (8.11%), and antibiotic inactivation (3.70%) were the most abundant resistance mechanism types in older pigeons (Figure 5D).

Further, the ARGs were categorized into 10 types: unclassified, tetracycline, sulfonamide, polymyxin, multidrug, macrolide–lincosamide–streptogramin, chloramphenicol, beta-lactam, bacitracin, and aminoglycoside. The gene distribution of ARGs in pigeon gut microbes is shown in the form of a histogram. The histogram of intestinal microbiota revealed that unclassified_sdiA, tetracycline_tetracycline-resistance protein, sulfonamide_sul1, polymyxin_arnA, multidrug_smeD, macrolide–lincosamide–streptogramin_macB, chloramphenicol_floR, beta-lactam_class C beta-lactamase, bacitracin_bacA, and aminoglycoside_aph(6)-I were the most abundant ARG types in both young and older pigeon gut microbiota (Figure 5E). In addition, the heatmap of ARG types indicated that the abundance of tetracycline was higher than that of other types in young pigeons, while the abundance of multidrug was higher than that of other types in older pigeons (Figure 5F). The heatmap of ARG subtypes showed that the abundance of tetracycline-tetW, aminoglycoside-aadD, and tetracycline-tetL was much higher than that of other subtypes in young pigeons, while the abundance of multidrug-acrB, multidrug-mdtF, and multidrug-transporter was much higher than that of other subtypes in older pigeons (Figure 5G).

## 4. Discussion

Pigeon breeding is an emerging specialized industry in China, which began in the 1980s. Due to the nutrient richness and fat lowness of pigeon meat and eggs, pigeons are reared as another new choice for meat and egg consumers, and they have a wide market in China. It has been reported that the gut microbiota constitutes the intestinal microecological system, maintaining a relatively stable state through interactions with the host, and is closely related to the health status of the body [27]. However, there is currently little research on the gut microbiota of pigeons because most gut microbes cannot be cultivated in the laboratory, which limits our understanding of gut microbial functions and hinders the healthy development of the pigeon breeding industry [28].

In this study, a metagenomics approach based on high-throughput sequencing was used to investigate the characteristics of the gut microbiota in young and older pigeons. We found that Bacillota, Uroviricota, and Pseudomonadota were the most abundant gut microbiota in both young and older pigeons. There are reports that Firmicutes, Bacteroidetes, and Proteobacteria are the dominant microbial phyla in chickens and many other bird species, such as white cranes, swan geese, Cygnus, Egretta garzetta, and Cygnus atratus [29,30,31]. It was reported that Bacillota had an antagonistic effect on the growth of pathogenic bacteria and can promote the growth of probiotics during the short breeding period of meat pigeons [32]. Uroviricota may play an important role in immune response induction and protection against other pathogens in pigeons. Pseudomonas helps pigeons degrade cellulose and produce various antibiotic compounds, which can effectively curb diseases caused by pathogenic bacteria and fungi [33]. It was known that in long-term artificial breeding and breeding, the intestinal microbial community of domestic pigeons may be more adapted to artificial feed and breeding environments, its flora structure may be relatively simple, and its resistance to certain pathogens may be weak [34]. In general, Firmicutes, Proteobacteria, and Actinobacteria are the dominant microbial phyla in chickens and other birds. The gut microbial composition and characteristics of pigeons differ from those of other birds due to unique physiological traits, diets, and captivity [35]. Compared with other wild birds, the types and quantities of external microorganisms pigeons are exposed to may be different, which directly affects the composition of the gut microbiota. As a specific bird species, the physiological characteristics and genetic background of pigeons may determine the uniqueness of their gut microbiota. Additionally, the health status of pigeons, especially the occurrence of intestinal diseases, will significantly change the composition of their gut microbiota. It was also noted that during the feeding process, the abuse of antibiotics would destroy the balance of the gut microbiota of pigeons, resulting in a decrease in beneficial bacteria and an increase in harmful bacteria, further affecting the composition of the gut microbiota. The microbial diversity of young and older pigeons analyzed in this study showed certain differences. The content of Streptococcus, which belongs to Firmicutes, was significantly higher in young pigeons compared to older pigeons, while the content of Escherichia was significantly higher in older pigeons. Notably, the content of Enterococcus in the gut microbiota of young pigeons was higher than that in older pigeons. Enterococcus belongs to lactic acid bacteria, and probiotic enterococci can effectively inhibit intestinal pathogens and maintain intestinal health [36]. Compared with older pigeons, the higher content of Enterococcus and lower content of Escherichia in young pigeons (Figure 1D) might indicate that with age increasing, beneficial bacteria decrease and pathogens increase.

In recent decades, the threat of new antibiotic resistance to public health has emerged and spread globally. As one of the world’s largest producers and consumers of food chain animals, China faces public health safety issues caused by the abuse of veterinary antibiotics in animal food production. Studies have found that various antimicrobial drugs remain in animal feces [37]. The members of microbial ecosystems, such as bacteriophages, have an important impact on the spread of antibiotic-resistant genes. They not only directly affect the number of microorganisms but also indirectly promote or inhibit the spread of antibiotic-resistant genes by reshaping the spatial structure of microbial communities. The most common antibiotics found in the feces of edible animals in China are fluoroquinolones, followed by sulfonamides, tetracyclines, and MLS. Antibiotics were previously abused in the pigeon breeding industry to promote growth rates and prevent disease incidences [38]. As a vast reservoir of ARGs, several studies have highlighted the importance of the intestinal microbiome in pigeon health and performance [39]. Thus, it is important to investigate antibiotic resistance, ARGs, and their association with the host in the pigeon gut microbiome.

In this study, metagenomic sequencing was used to identify the presence and relative abundance of ARGs in young and older pigeons. A key advantage of the metagenomic approach is its capacity for unbiased cataloging of ARGs. However, there are some differences in results depending on specific purposes, ranging from the analysis of known ARGs to the discovery of completely novel ARGs [40]. The results showed that 142 ARGs were identified, including adeJ, mdtC, tet(M), and udg; the corresponding genes had the highest relative abundance in the pigeon gut microbiota. AdeJ is an inner membrane transporter protein associated with the resistance–nodulation–cell division (RND) type of efflux pump, which is found in tigecycline-resistant Acinetobacter baumannii. We matched each ARG to its corresponding antibiotic and concluded that the ARGs in the pigeon gut microbiota confer resistance to almost all major antibiotic classes commonly employed for agricultural use. In young pigeons, tetracycline resistance was most commonly found, while multidrug resistance was most abundant in older pigeons (Figure 5G). In recent decades, tetracyclines have been widely incorporated into animal feed to reduce disease incidence and increase growth rates. Although China banned the use of antibiotics as growth promoters in 2020, ARGs have already been deposited in microbial genomes. Rovira et al. found that discontinuing the use of tetracyclines on farms did not automatically reduce resistance [41]. Ma et al. reported that ARGs can be transmitted from livestock animals to humans and are widely spread across different environments [42]. In addition, bacterial taxonomic groups vary in their association with specific ARGs. In general, gram-positive bacteria often harbor ARG genes like methicillin and vancomycin. Gram-negative bacteria frequently carry ARG genes such as beta-lactams, aminoglycosides, and fluoroquinolones. Pseudomonas is often resistant to multiple antibiotics, including carbapenems and aminoglycosides. Further, it is reported that the microbiome and drug-resistant gene composition of wild pigeons are complex and diverse, posing a potential threat to public health and animal health. The composition of fecal microorganisms in wild pigeons is mainly affected by the geographical environment. Wild pigeons may carry and spread a variety of microorganisms, including potential pathogens. The composition of drug-resistant genes in wild pigeons may carry multidrug-resistant strains and drug-resistant genes, such as Klebsiella pneumoniae. These drug-resistant genes may be transmitted between microorganisms through mobile elements such as plasmids, increasing the risk of spreading drug resistance. Specific drug-resistant genes, such as mcr1, have been found in avian pathogenic Escherichia coli and may be transmitted from poultry to humans and other animals. Hence, the observation and research of antibiotic resistance genes not only has far-reaching biological significance but also has an important impact on the economy. Strengthening relevant research and exploring effective control strategies are of great significance for protecting human health and promoting sustainable economic development, and careful use of antibiotics is recommended to prevent the increase and transfer of key ARGs from farms.

Pigeons are poultry products sold within a short period, so the antibiotics issue requires special attention. Excessive use of antibiotics not only causes antibiotic residues but also leads to antibiotic resistance. Therefore, in the management of pigeon breeding, it is essential to strengthen deworming and disinfection, maintain environmental hygiene, and pay special attention to drug management.

## 5. Conclusions

In conclusion, a metagenomic analysis was performed to investigate the gut microbial communities and ARGs in young and older pigeons. The results explained the differences in the dominant gut microbiota and functional compositions between young and older pigeons from different regions in China. Additionally, 142 ARGs conferring multidrug resistance, tetracycline, and aminoglycoside resistance were identified, representing more than 70% of the fecal resistomes; the most abundant gene in young pigeons was tetracycline-tetW, while in older pigeons, it was multidrug-acrB. Additionally, the highest abundance of resistance genes in older pigeons indicates a high level of antibiotic resistance. Our study helps to understand pigeon gut microbiota and antibiotic resistomes and contributes to knowledge-based sustainable pigeon meat production.

## Data Availability

The raw metagenomic sequencing data generated in this study have been deposited in the Genome Sequence Archive at the National Genomics Data Center, China National Center for Bioinformation/Beijing Institute of Genomics, Chinese Academy of Sciences (https://ngdc.cncb.ac.cn/gsahuman, Accession No. CRA021152, accessed on 1 October 2024). All datasets presented in this study are included in the article material or available with the corresponding author if needed. All R packages are available online, as described in the Methods.

## Supplementary Materials

The following supporting information can be downloaded at: https://www.mdpi.com/article/10.3390/biology14010025/s1, Figure S1: Pathogen Host Interactions (PHI) characteristics of gut microbiota between young pigeon and older pigeon; Figure S2: GraPhlAn visualization of hierarchical annotation between young pigeon and older pigeon samples; Table S1: Metagenomic sequencing information; Table S2: Gene alpha diversity analysis; Table S3: The information of the top 10 abundant pathogens in young pigeons; Table S4: The information of the abundant pathogens in older pigeons.
