# Supplementary material for: Metagenomic Insights into Pigeon Gut Microbiota Characteristics and Antibiotic-Resistant Genes
Source: Biology (Basel). 2025 Jan 1;14(1):25. doi: 10.3390/biology14010025 (PMC11763083; doi:10.3390/biology14010025)
Supplement: Supplementary file 1 [file biology-14-00025-s001.zip › biology-3320390-supplementary.pdf]

Metagenomic Insights into Pigeon Gut Microbiota Characteristics and Antibiotic-Resistant Genes

Wei Dai [1,†], Haicong Zhu [1,†], Junhong Chen [1], Hui Chen [1], Dingzhen Dai [1] and Jian Wu [2,*]

Supplementary Information

Table S1. Metagenomic sequencing information.

Sample	Total reads	Total bases (bp)	Q20 bases ratio	Clean reads	Clean reads ratio	N50	GC content
YP	38,220,082	5,733,012,300	94.57%	36,035,534	94.28%	1,786	41.66%
OP	40,093,458	6,014,018,700	94.03%	37,528,722	93.60%	1,653	51.07%

Table S2. Gene alpha diversity analysis.

Sample	Observed	Chao1	ACE	Shannon	Simpson	InvSimpson	Coverage
YP	68,311	69,100.96	69,477.81	7.61	0.99	144.18	0.999,8
OP	79,975	81,253.26	81,678.94	8.91	1.00	2,035.15	0.999,8

Table S3. The information of the top 10 abundant pathogens in young pigeons.

PHI gene	Pathogen	Disease	Host	Host species	Gene function
CBJ02741	Escherichia coli	Urinary tract infections	Rodents	Mus musculus (house mouse)	Secreted metalloprotease and surface associated lipoprotein
ADE32434	Streptococcus suis	Meningitis	Even-toed ungulates	Sus scrofa (pig)	Inosine-5'-monophosphate dehydrogenase
ABP90420	Streptococcus suis	Meningitis, septicemia and streptococcal toxic shock syndrome	Rodents	Mus musculus (house mouse)	Central tRNA-modifying GTPase
AAK34199	Streptococcus pyogenes	Skin lesions	Rodents	Mus musculus (house mouse)	Phosphoenolpyruvate-protein phosphotransferase
AAL00050	Streptococcus pneumoniae	Pneumococcal pneumonia	Rodents	Mus musculus (house mouse)	ABC transporter ATP-binding protein - spermidine/putrescine transport

Table S4. The information of the abundant pathogens in older pigeons.

PHI gene	Pathogen	Disease	Host	Host species	Gene function
CBJ02741	Escherichia coli	Urinary tract infections	Rodents	Mus musculus (house mouse)	Secreted metalloprotease and surface associated lipoprotein

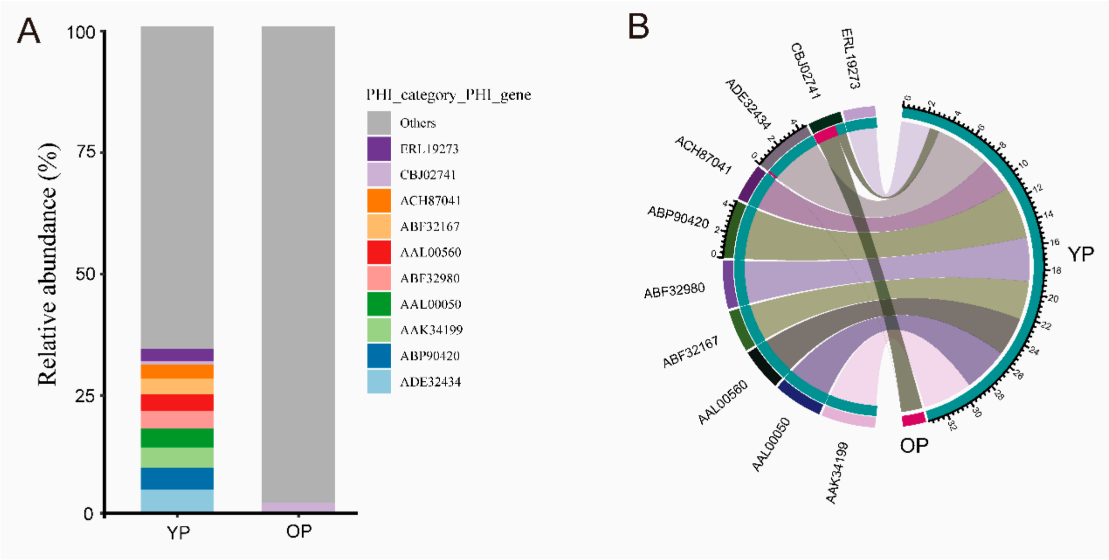

Figure S1. Pathogen Host Interactions (PHI) characteristics of gut microbiota between young pigeon and older pigeon. (A) Relative abundance of PHI gene. (B) Circos plot of relative abundance of PHI gene classification. The right side of the circle indicates group information, the left side indicates PHI classification information; the Outer circle is the ideogram scale of the distribution of unique genes, and the inner circle represents different groups.

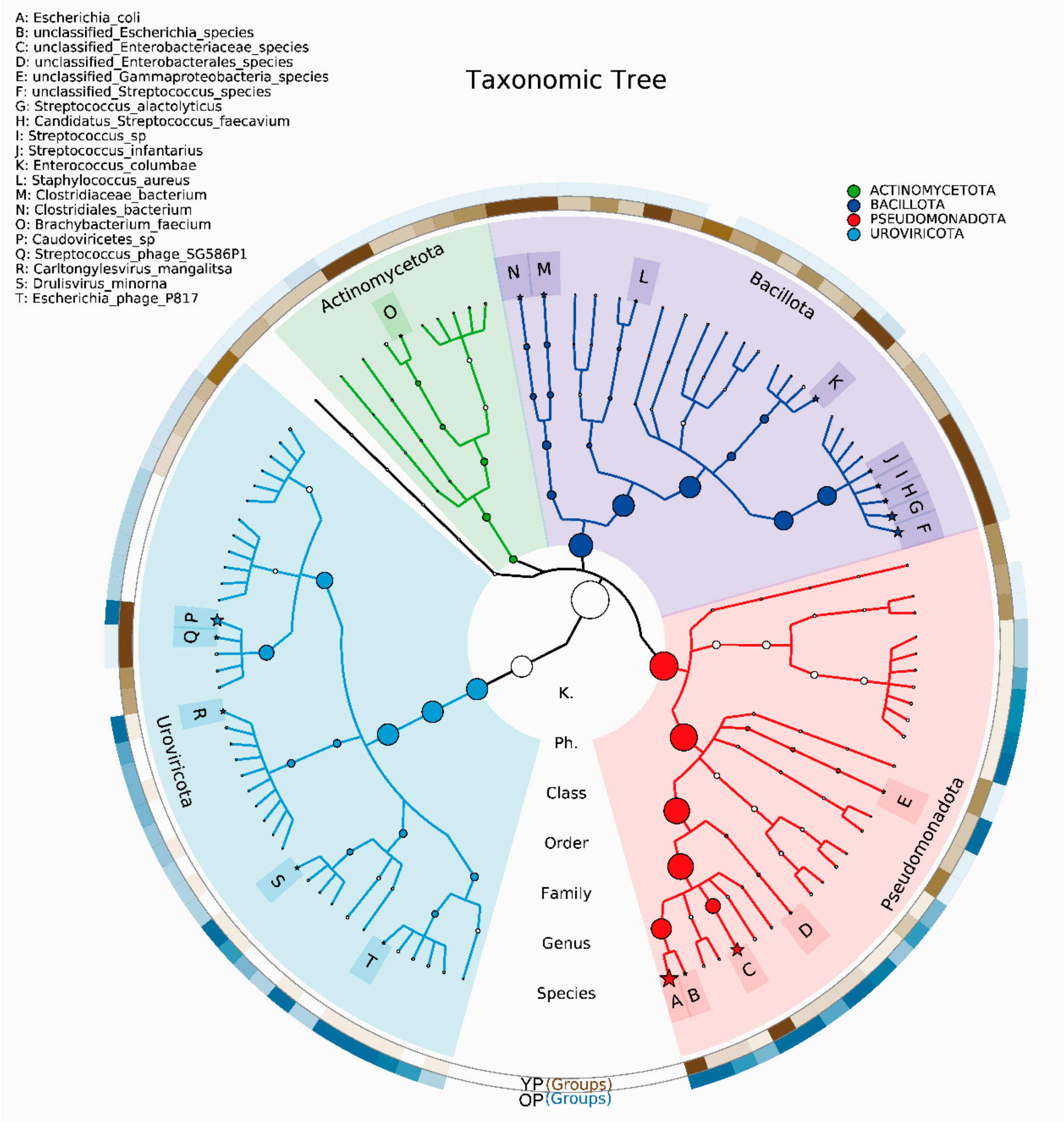

Figure S2. GraPhlAn visualization of hierarchical annotation between young pigeon and older pigeon samples. The center represents the evolutionary classification tree of the top 100 species in abundance, and the gates corresponding to the top 20 species in abundance (marked with asterisks) are marked with different colors. The size of the circle and the asterisk represents the abundance. The outer ring is a heat map, each ring is a sample, and each sample corresponds to a color. The color depth varies with the abundance of the species.
